# ANARI: A 3-D Rendering API Standard

**DOI:** 10.1109/mcse.2022.3163151

**Published:** 2022-03-30

**Authors:** John E. Stone, Kevin S. Griffin, Jefferson Amstutz, David E. DeMarle, William R. Sherman, Johannes Günther

**Affiliations:** University of Illinois at Urbana-Champaign, Urbana, IL, 61801, USA; NVIDIA, Santa Clara, CA, 95051, USA; NVIDIA, Santa Clara, CA, 95051, USA; Intel Corporation, Folsom, CA, 95630, USA; National Institute of Standards and Technology, Gaithersburg, MD, 20899, USA; Intel Corporation, 85579, Munich, Germany

## Abstract

ANARI is a new 3-D rendering API, an emerging Khronos standard that enables visualization applications to leverage the state-of-the-art rendering techniques across diverse hardware platforms and rendering engines. Visualization applications have historically embedded custom-written renderers to enable them to provide the necessary combination of features, performance, and visual fidelity required by their users. As computing power, rendering algorithms, dedicated rendering hardware acceleration operations, and associated low-level APIs have advanced, the effort and costs associated with maintaining renderers within visualization applications have risen dramatically. The rising cost and complexity associated with renderer development creates an undesirable barrier for visualization applications to be able to fully benefit from the latest rendering methods and hardware. ANARI directly addresses these challenges by providing a high-level, visualization-oriented API that abstracts low-level rendering algorithms and hardware acceleration details while providing easy and efficient access to diverse ANARI implementations, thereby enabling visualization applications to support the state-of-the-art rendering capabilities.

Three-dimensional visualization is a broad field, experiencing innovation in visual computing technology over decades and spanning countless domains, such as design engineering, computational science, and artistic creativity. Considerable rendering software has been produced through its storied history, both to directly render effective, state-of-the-art visualizations, and to enable new visualization workflows that serve user needs.

As computing capabilities continue to grow at staggering rates, so has the complexity of the software systems used to harness them. The rapid evolution of hardware architectures combined with increased software complexity, has led to reduced interoperability. In order to tame software complexity, cross-industry application programming interface (API) specifications exist to let developers use common interfaces with multiple vendor implementations, while leaving vendors room to innovate within their implementations. Open standards provide *interoperability by design,* rather than limited ex post facto software compatibility. The analytic rendering interface (ANARI) standard offers interoperability, and aims to bring innovative 3-D rendering engines under a portable API for developers to leverage in their applications.

## CONTEXT: WHAT IS 3-D VISUALIZATION?

The state-of-the-art 3-D rendering techniques that approximate the physics of light transport are based on the combination of sophisticated, hardware-optimized algorithms with massively parallel computing hardware that often includes dedicated logic for acceleration of the most performance-critical operations. Depending on the needs of the application, approximations can be used to replace costly path tracing, with commensurate decreases in required computing performance, and attendant reductions in power consumption, which is important for laptops and other mobile platforms. In other cases, such as accurate rendering of architectural lighting designs, and rendering of car headlight designs, approximations would be unacceptable, thus greater parallelism, hardware resources, and hardware-accelerated rendering are required. The challenges posed by the growing size and complexity of data to be visualized, the desire for greater image fidelity, interactivity, realistic physically based rendering, and interest in immersive visualization techniques are all examples of the kinds of competing demands that increase the complexity and cost of renderer development for today’s applications.

## ANARI POSITIONING

**FIGURE 1.**
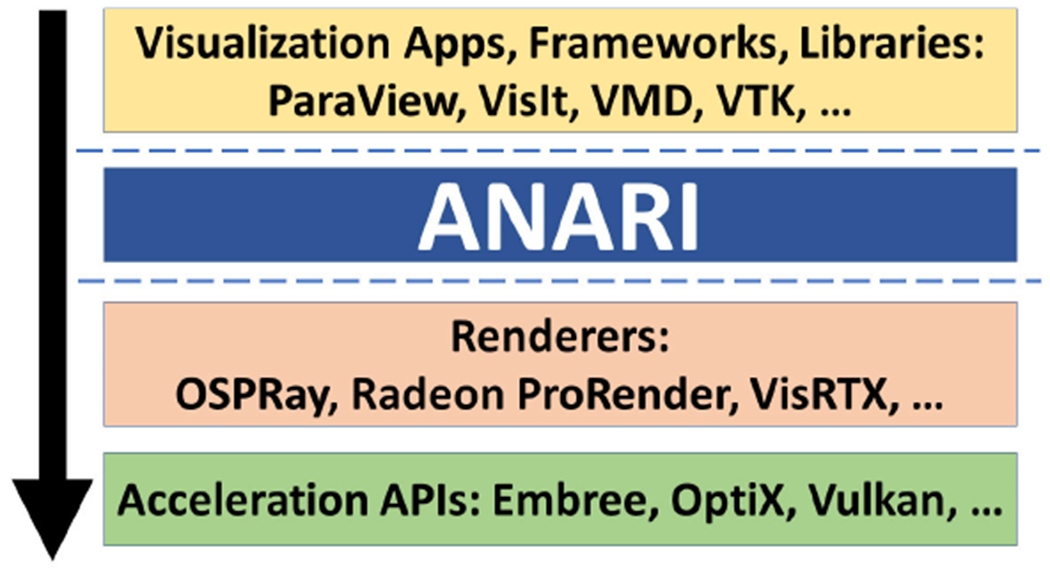
ANARI’s position between applications, renderers, and supporting hardware-optimized acceleration APIs. Note that lower positioned APIs and libraries tend to be less portable and require more expertise to fulfill application needs.

**FIGURE 2.**
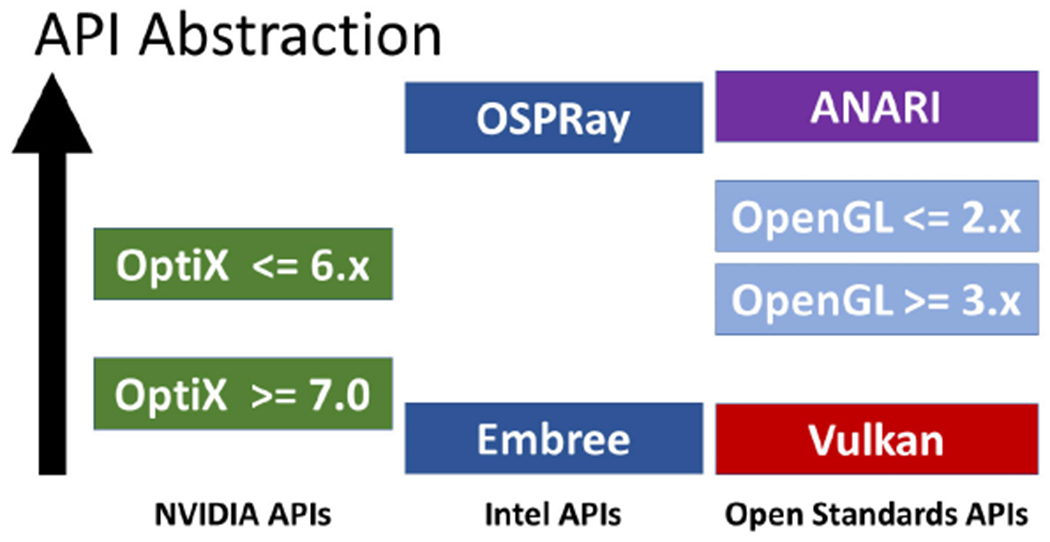
Qualitative comparison of the level of abstraction provided by exemplary rendering APIs that are widely used by visualization applications.

The ANARI API enables developers to build a scene description to generate imagery, rather than specifying the details of the rendering process, providing simplified application development, and cross-vendor portability to diverse rendering engines. ANARI is positioned as a high-abstraction rendering API that encompasses renderers built on both rasterization and state-of-the-art ray tracing methods, and supports rendering styles that range from stylized or nonphotorealistic schematic renderings to photorealism and complete physical correctness. Figure 1 shows ANARI’s role in-between visualization software and the ecosystem of renderers and supporting APIs. Figure 2 shows a qualitative comparison of the abstraction provided by ANARI relative to other industry APIs used to develop visualization and design applications.

**FIGURE 3.**
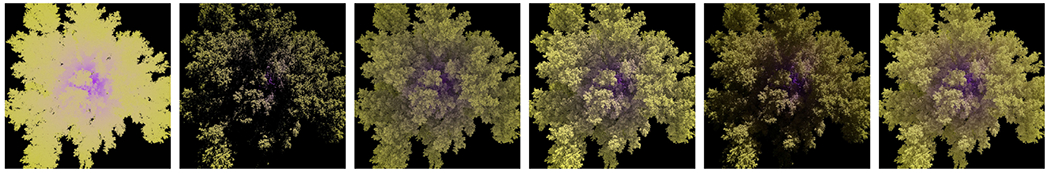
Comparison of lighting techniques for a complex and crowded visualization of the results of a diffusion-limited aggregation simulation. All renderings used ANARI with the VisRTX back-end and different lighting parameters. The lighting techniques, from left, are: ray casting of surface color, directional lighting and shadows, ambient occlusion lighting, directional lighting with ambient occlusion, directional lighting with path traced indirect lighting, and directional lighting with ambient occlusion and path traced indirect lighting.

Recent progress in rendering technology, especially the introduction of real-time ray tracing, promises to fundamentally impact markets far beyond media and entertainment uses. For example, scientific visualization applications not only benefit from the physically accurate generation of images, but also from important visual cues afforded by ray tracing; cues that provide an intuitive understanding of complex scenes and the diverse workflows built around them. Figure 3 illustrates how the use of advanced lighting and shading techniques can help elucidate geometry-dense scenes with complex 3-D structure. However, these powerful capabilities come at the cost of increasing developer responsibility. While low level APIs, such as Vulkan and its ray tracing extensions, have been standardized to provide some abstraction of the recent rendering hardware and software developments, commercial software vendors and open-source efforts still need to develop their entire rendering code on top of these low-level APIs. While this is core business for applications focused on rendering, there is a broad range of applications for which rendering is just a necessary technique to achieve an end. For these applications, developers need a lower barrier of entry to be able to take full advantage of new and emerging rendering technologies, such as real-time ray tracing.

The goal of ANARI is to provide a high-level, platform-independent API to simplify development of visualization applications leveraging the full potential of modern rendering capabilities. Rather than specifying details of the rendering process, ANARI describes the relationship of the objects to be rendered and leaves the details of rendering to the underlying implementation. Unlike more general scene graph APIs, ANARI focuses primarily on rendering operations and leaves other domain-specific scene operations in the hands of the application itself. Scene graphs can be implemented using ANARI to handle their rendering work. ANARI renderers are free to incorporate technologies, such as AI denoising, and to expose new ANARI extensions, e.g., those that add new geometric primitives, load custom shaders, or provide enhanced efficiency with other APIs.

## PAST PERSPECTIVE: A LOOK AT SCIENTIFIC VISUALIZATION’s HISTORY LEADING TO ANARI

While ANARI is designed to interface 3-D rendering engines to applications from practically any domain, reviewing the historical trends in scientific visualization provides a good perspective on why ANARI was created.

Software tools for scientific visualization have historically been tightly coupled to the rendering hardware and software of the era in which they were written. The visualization features and rendering approaches embodied in these tools were carefully designed to provide the best combination of visual insight, ease-of-use, and performance, on the hardware of their time.

Early visualization tools written prior to the widespread availability of commodity rasterization accelerators often used software-based rasterization or raycasting techniques. These tools typically employed entirely custom-written internal renderers to achieve the required degree of interactivity. An excellent example of this approach is RasMol, which used clever sphere drawing algorithms to achieve molecular graphics performance levels that outperformed most hardware-accelerated rasterization approaches until circa 2000.^1^

The arrival of commodity hardware-accelerated rasterization began in earnest in the early 1990s. Silicon Graphics’ proprietary IRIS GL API became more popular than all other proprietary and industry standards of the time due to its ease of use, but it was not well suited to diverse hardware, and it offered no abstraction layer for missing hardware capabilities. VMD, a widely used molecular visualization tool, was one of many originally written the for IRIS GL.^2^ Realizing the need for an API that improved upon IRIS GL, by eliminating functionality unrelated to rasterization (windowing, mouse input, etc.) a better suited API for cross-platform standardization was developed by Silicon Graphics and its collaborators, replacing IRIS GL with OpenGL.

When Silicon Graphics released the OpenGL API and it gained widespread adoption, a new generation of scientific visualization tools was born. OpenGL provided improved rendering abstractions with greater generality, and a rich set of core features. By the early 2000s, even gaming-oriented graphics boards were capable of accelerating OpenGL in hardware. The widespread availability of OpenGL across hardware ranging from PCs, to workstations, all the way up to supercomputers made it the dominant API underpinning most scientific visualization applications. This led programs like VMD that had originally been written in IRIS GL to be redesigned for OpenGL. OpenGL became the gateway that enabled many scientific visualization and CAD applications to run on PC hardware for the first time.

Early OpenGL relied on a fixed-function rendering pipeline, and visualization applications frequently used similar techniques for visualizations of particular types of data, with the same overall “look.” Over time, major OpenGL API advances replaced fixed-function features with the current higher performing retained mode interface and flexible programmable shading pipeline architecture, leading to diversification of shading capabilities and techniques. Visualization applications too went through commensurate changes and advances, taking advantage of per-pixel lighting, and sophisticated procedural geometry rendering techniques, such as ray casting of imposter spheres and cylinders within custom-written fragment shaders, and high-fidelity volume ray casting, all while achieving, in some cases, an order of magnitude increase in rendering performance.^3,4^ The new rendering capabilities improved scientific insight, visual fidelity, and performance, leading visualization applications to continue revising their internal renderers to exploit them.

As a result of the increasing complexity of rasterization hardware and OpenGL features, the associated software development complexity and “buy-in” costs for scientific visualization applications to remain abreast of the latest rendering techniques have risen significantly. At the same time, state-of-the-art rasterization APIs, such as Vulkan, the heir and descendant of OpenGL, have become more lightweight and minimalistic, and place both more control and more responsibility in the hands of the application—in exchange for performance and flexibility. The value provided by state-of-the-art OpenGL and Vulkan APIs comes with a high buy-in cost, and scientific visualization application developers are left to write increasingly complex rendering code.

Due to the increasing computational capabilities of CPUs and GPU accelerators and hardware acceleration of fundamental ray tracing algorithms, fully interactive ray tracing is now possible for many important visual effects, and scientific and technical visualization workloads. Since ray tracing, and path tracing in particular, rely heavily on Monte Carlo integration and stochastic sampling techniques, a former barrier to their adoption for challenging scenes had been the necessity to obtain images with acceptably low residual image grain or noise. Advances in the application of so-called AI denoising techniques to the results of ray tracing and path tracing techniques reduce or eliminate the necessity for rendering engines to produce completely converged images. The associated increase in the quality and frame rates of live renderings makes them practical for use in a much broader range of contexts.

With the advent of high-performance ray tracing engines,^5,6^ visualization applications began to take advantage of them. For example, the VTK library as well as ParaView and VisIt, two high-profile visualization applications that incorporate VTK, embedded interactive ray tracing capabilities over a number of years, beginning with the University of Utah’s Manta Ray tracer in 2010 and then Intel’s OSPRay and NVIDIA’s VisRTX libraries in 2016 and 2019, respectively. Similarly to the update of VTK’s OpenGL rendering engine, these changes brought about compelling new features, but each required several months of core developer time to achieve.

The ANARI API provides a much higher level of abstraction than APIs, such as OpenGL or Vulkan, as indicated by the abstraction comparison shown in Figure 2. Rather than abstracting hardware pipelines, ANARI abstracts rendering altogether, thereby completely eliminating the need for the scientific visualization application developer to write a renderer. While the incorporation of ANARI-based rendering into an application requires development time and effort, the high-level abstractions it provide require much less code, and it is much simpler to obtain high-performance and high-fidelity output with ANARI than with low-level APIs. The ANARI API allows applications to exploit state-of-the-art rendering algorithms and hardware acceleration, freeing application developers to focus on core data analysis and visualization algorithms, graphical representation, and scene generation.

### Visualization Application Vignette: VisIt

VisIt is a distributed, parallel scientific visualization, and graphical analysis tool for data defined on 2-D and 3-D meshes.^7^ VisIt’s rendering capabilities relied on two main rendering APIs; VTK for data models and plotting, and OpenGL for creating custom plots. Once OpenGL 2.0 was published in September 2004, two parallel efforts were undertaken to allow VisIt to use modern OpenGL’s programmable shaders. The first effort was to upgrade VTK once it integrated modern OpenGL features, and the second effort involved updating VisIt’s custom plots to use modern OpenGL’s programmable shaders. The amount of effort involved in this process would have been cut in half, at a minimum, for its 3-D surface and volume plots (e.g., pseudocolor, subset, and volume) if a higher level API like ANARI was available.

At its peak complexity, VisIt had eight different types of renderers for visualizing 3-D surface and volume data. Six were used for volume rendering (RayCasting: Compositing, RayCasting: SLIVR, OSPRay, Splatting, 3-D-Texture), and two for surface rendering (VTK, OpenGL).^8^ This means that VisIt developers had to learn the inner workings of multiple renderers and, absent documentation, track down the original implementers to understand design decisions and more complicated coding dependencies. ANARI’s goal is to alleviate developers of this type of headache by providing a common interface for all of these renderers and additional renderers currently used in other domains. VisIt developers using ANARI will now only need to maintain a minimum amount of code for loading and choosing the desired renderer. The maintenance and implementation of the renderers will be the responsibility of their implementers. Using ANARI, VisIt will be able to add advanced and hardware-based rendering capabilities to it is 3-D surface and volume plots by leveraging the additional renderers available through the ANARI interface.

### Visualization Application Vignette: VMD

VMD is a widely used molecular visualization tool that specializes in the display and analysis of molecular dynamics simulations.^2^ VMD was originally developed using Silicon Graphics IRIS GL, was ported to OpenGL in 1998, and OpenGL 2.0 in 2004. Since its inception, VMD supported ray tracing as an offline rendering technique for generating publication quality figures. It was later adapted to make use of hardware-optimized ray tracing with a custom-written internal rendering engine that supported the curved geometric primitives and other scene content heavily used in molecular visualization.^5,9^ Later developments have added fully interactive progressive ray tracing to the built-in rendering engines, support for more hardware-optimized ray tracing engines,^6^ and support for instancing and other new features used for cell-scale visualization.^10^ Today, VMD contains several custom-written internal renderers based on OpenGL, EGL, OptiX,^5^ and Tachyon, and it incorporates OSPRay^6^ internally with a renderer subclass wrapper. It is clear that there are great opportunities for ANARI to initially augment, but ultimately to completely replace the large collection of custom-written and internally adapted renderers within VMD, thereby reducing the cost of ongoing renderer development and maintenance, and enabling it to more easily take the full advantage of state-of-the-art rendering algorithms and hardware acceleration technologies.

### Visualization Application Vignette: VTK and ParaView

ParaView^11^ is a scalable, general purpose visualization environment that is built from and developed in tandem with VTK by Kitware Inc, along with an open-source developer community.^12^ With the exception of application level control and parallel depth compositing, ParaView’s rendering infrastructure is entirely implemented at the VTK level. VTK’s releases currently have three rendering interfaces, OSPRay, VisRTX, and OpenGL, where OpenGL is primary and OSPRay and VisRTX share a common control layer. Each interface interprets the same renderable scene state, but issues different commands to drive the corresponding external rendering engine. A suite of continuous integration regression tests validates rendering correctness of all three and ensures close correspondence between base visual appearances for VTK’s vast set of drawable items, including surface and volume rendering of a number of core data structures.

VTK’s implementation for OSPRay and VisRTX has served as a working prototype for ANARI integration into VTK and ParaView throughout ANARI’s standard definition timeline. Figure 10 shows an example ParaView visualization created using a developmental version of the example back-end device included in the ANARI-SDK. We anticipate that VTK and ParaView’s initial interface to ANARI will be closely aligned with VTK’s existing ray-traced rendering code.

## ANARI API OVERVIEW

ANARI is a C99 API, which follows the practice of most other Khronos API standards and has advantages, such as the ease of integration with other programming languages (e.g., Python) and familiar tooling in industry.

The API is object oriented, where objects represent actors in the rendering process, such as cameras, renderers, and the visible entities, such as geometries and volumes to be rendered in the scene. These objects are parameterized with string-value pairs using a fixed set of types that ANARI expresses, which can include other object handles.

Similarly, objects can publish property values that applications use to introspect information from the implementation, such as the world-space bounds of a triangle mesh. These properties draw from the same set of types used to encode parameters.

The API is implemented by software device objects, which are passed as the first argument to each ANARI function. Devices encapsulate the implementation of the entire API and use the same parameter and property semantics like all other objects for configuration and status monitoring. It is worth noting that ANARI devices are software constructs: implementations choose what hardware resources their renderers will use, where configuration of such resource usage is done through parameters specific to the device.

Some of the most fundamental objects in ANARI are arrays. Array objects represent multiple data array semantics: memory ownership between the device and application, element type, data update mechanics, and array dimensionality. Arrays are flexible to operate in the best way for the application’s needs: applications can share memory directly with an ANARI device to minimize memory overhead, or instead let the device allocate memory for increased control and performance.

Performance is an important aspect of interactive 3-D applications, where ANARI permits device implementations to take advantage of asynchronous, or non-blocking, API semantics to maximize throughput. The ANARI API is defined in such a way that applications do not have to be blocked by long-running frames, allowing them to keep other parts of the application still interactive, such as a graphical user interface.

Finally, the design used to create objects, parameterize them, and read their properties is open to extension. Extensions come in two forms: 1) core extensions are optional features, which the ANARI specification defines, 2) and vendor extensions are features not in the specification that could later be standardized as a core extension. Using strings to identify object types, parameters, and properties allows core features, core extensions, and vendor extensions to all use the same API calls, which keeps the ANARI API itself a small, manageable size. Since ANARI APIs operate at a high level of abstraction, the overhead from string operations is negligible in practice.

## ANARI FRONT-END LIBRARY AND SDK

ANARI application developers and device implementers meet via a common front-end library used to map ANARI’s API to back-end devices. This library, along with helpful tools surrounding the API itself, is typically referred to as the “ANARI SDK,” which carries numerous advantages for those both above and below the API.

First, having a common SDK reduces boilerplate code that occurs with API standards that force implementers to ship the API’s function prototypes. This reduces the implementation burden for shipping implementations, and eliminates confusion by having only one place to obtain the headers needed to use ANARI.

Second, ANARI code to ease device implementation can be shared by vendors, as well as additional utilities for users, such as a C++ binding layer to add improved C++ type safety to the API.

Finally, ANARI’s SDK enables the injection of runtime tools that help application developers to find commonly occurring errors. As the standard was developed, device-agnostic debugging and tracing tools to validate the API were collaboratively produced to confirm key implementation choices and assist early exploratory usage. These tools and others continue to mature as industry interest grows and more implementations emerge.

## ANARI EXAMPLE CODE

**FIGURE 4.**
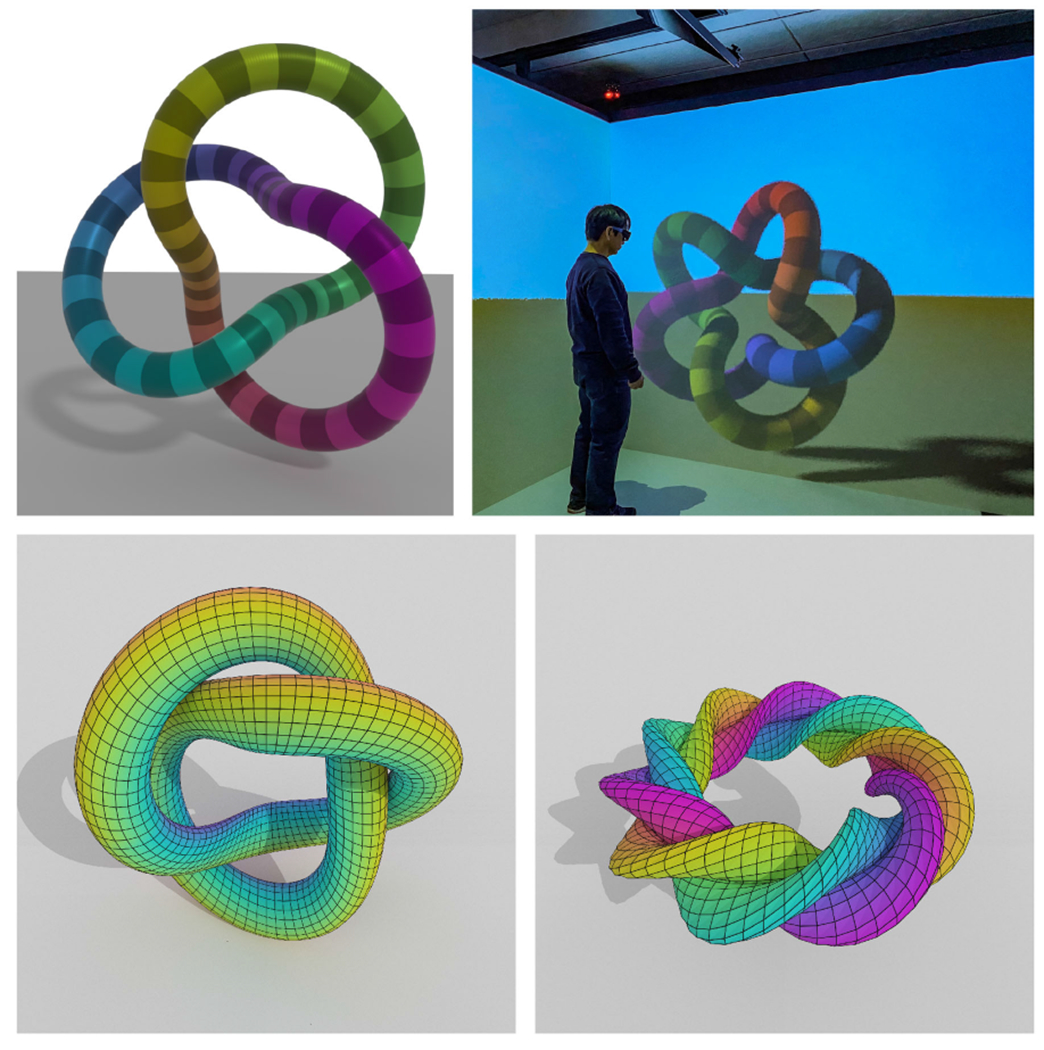
ANARI renderings of knots (top: OSPRay, using sphere primitives) and parametric surfaces (bottom: VisRTX, using triangle meshes and cylinder primitives) produced using early ANARI back-end renderer implementations, combining directional lights, ambient occlusion, and path tracing. ANARI knot example (top right) running with FreeVR in a three-wall CAVE at NIST. Examples are provided with the publicly available ANARI SDK and renderers.

To demonstrate the level of abstraction and ease of use of the ANARI API, we have shown the ANARI-specific parts of the knot in Figure 4, in Figures 11–13 omitting unnecessary details. The first ANARI code example shown in Figure 11 demonstrates a simple approach for loading an ANARI back-end library to instantiate an ANARI “device” from it, followed by using the device to create a perspective camera and set several of its parameters. Already in this short example, we can see the beginnings of key programming patterns that are common to the use of all of the ANARI APIs. The sequence of steps that create the camera object and prepare it for use are generally representative of the way most ANARI objects are created, parameterized, and used. Some ANARI object creation APIs accept string names of the particular subtype being created—in this case a “perspective” camera. The optional and required parameters associated with the newly created object subtype are then set with subsequent anariSetParameter calls.

When the object has been completely specified and no further changes will be made before the next frame, anariCommit tells the ANARI back-end device that it can finalize the object for rendering. Parameters set on an object are not used in the next frame until the application calls anariCommit on the object. This lets applications transition an object from one configuration to the next without the need to deal with intermediate, invalid object parameterizations.

Figure 12 shows the use of ANARI APIs to bind arrays of data as per-vertex coordinates and colors as input to an ANARI spheres geometry, to ultimately end up in an ANARI surface object. This example also shows how the anariRelease API is used to indicate when the calling application no longer needs an ANARI object handle. This gives the device implementation the freedom to free any resources no longer used by that object, including the object itself should no other objects be referencing it.

The final ANARI example shown in Figure 13 demonstrates a complete sequence of the APIs used to fully describe a scene by: 1) instantiating materials; 2) assigning them to geometric surfaces; 3) to create, configure, and bind a renderer; and finally 4) to create, configure, and finalize the ANARI frame, which references everything involved in creating the final image.

## EXAMPLE VISUALIZATIONS

During development of the ANARI API specification, the working group developed multiple prototype rendering device and application implementations that spanned both a diversity of hardware platforms (both CPU- and GPU-based) and rendering techniques (both rasterization- and ray tracing-based) to ensure that the design of ANARI APIs was closely coupled with actual implementation experience, as well as experience on the application development and debugging side. Here, we show a few exemplary visualization scenes that were used as early test cases when coupling several popular visualization tools to early ANARI device implementations.

**FIGURE 5.**
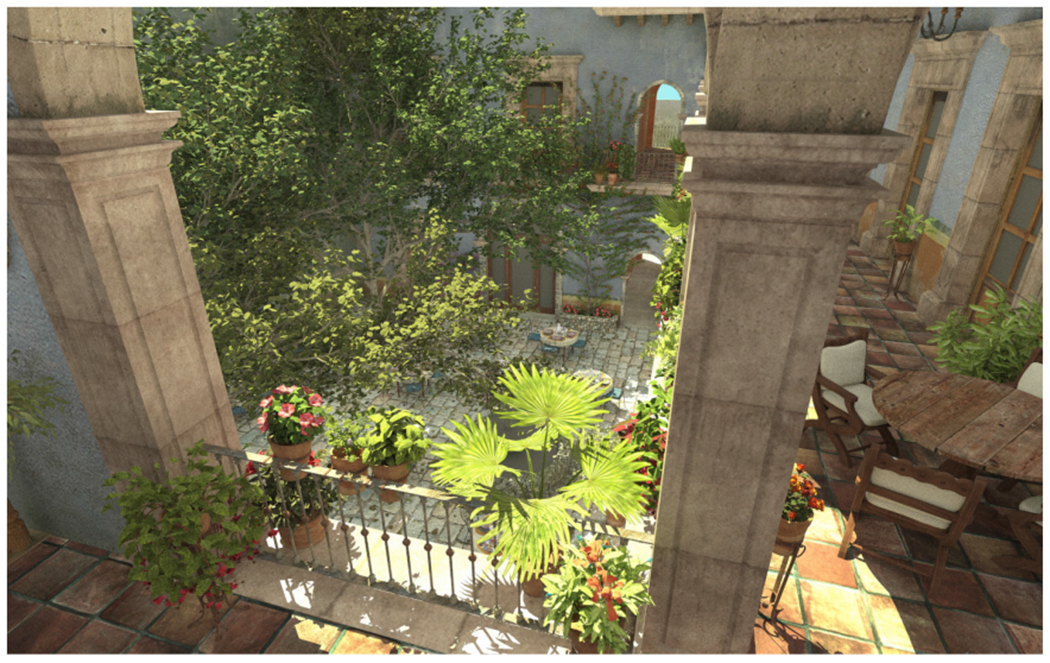
ANARI VisRTX path traced rendering of the San Miguel scene © Guillermo M. Leal Llaguno (https://casual-effects.com/data).

Figure 4 shows the output of two example ANARI applications that plot knots and parametric surfaces using ANARI’s APIs for rendering quad, triangle, sphere, and cylinder geometry subtypes using OSPRay and VisRTX back-ends. Figure 5 shows an ANARI rendering of the San Miguel scene using the developmental VisRTX back-end, highlighting ANARI’s support for image mapped texturing of surface geometry, and other features, e.g., as needed for architectural visualization, industrial design, entertainment, and similar application domains more broadly.

One exciting aspect of the industry’s movement toward real-time rendering with advanced algorithms is in bringing these capabilities to virtual reality displays. Depending on the style of VR, rendering frame rates of 90 Hz or more are often recommended. In most cases, this can be a difficult goal to attain, though with CAVE and fishtank style VR displays, lower frame rates can be acceptable. ANARI allows the user to choose the back-end that gives the best rendering performance for a particular immersive visualization. We have implemented several ANARI test applications that use the FreeVR library, which handles the head tracking, and calculates the camera parameters that ANARI uses to create a user-perspective view of the scene, as shown in Figure 4 (top right).

**FIGURE 6.**
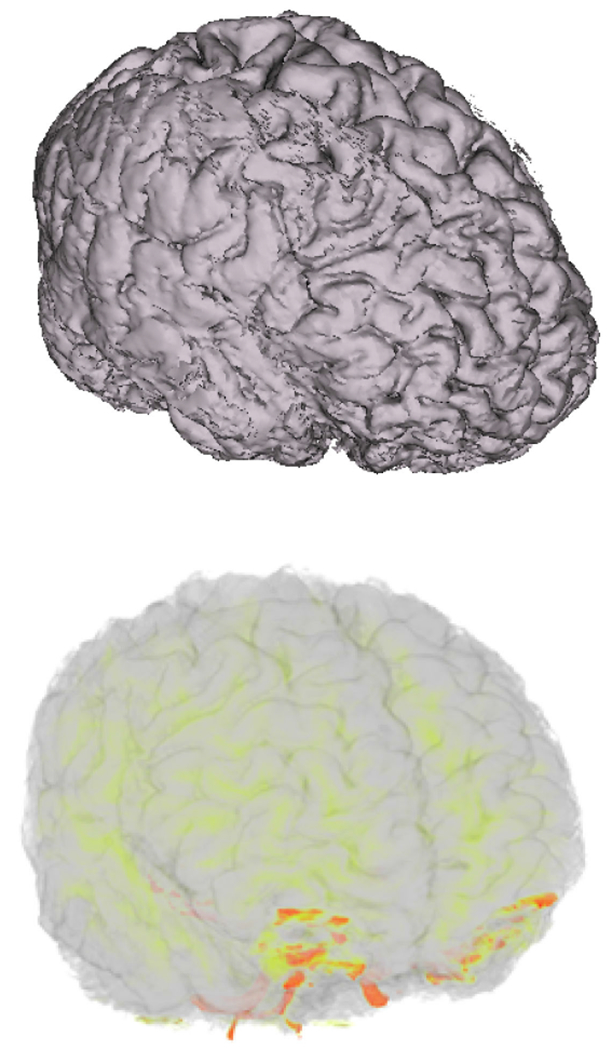
Human brain MRI dataset courtesy of the Mayo Clinic rendered in VisIt. (Top) Surface rendering of the MRI dataset using VisIt’s pseudocolor plot. (Bottom) Volume rendering done using ANARI with the VisRTX back-end.

**FIGURE 7.**
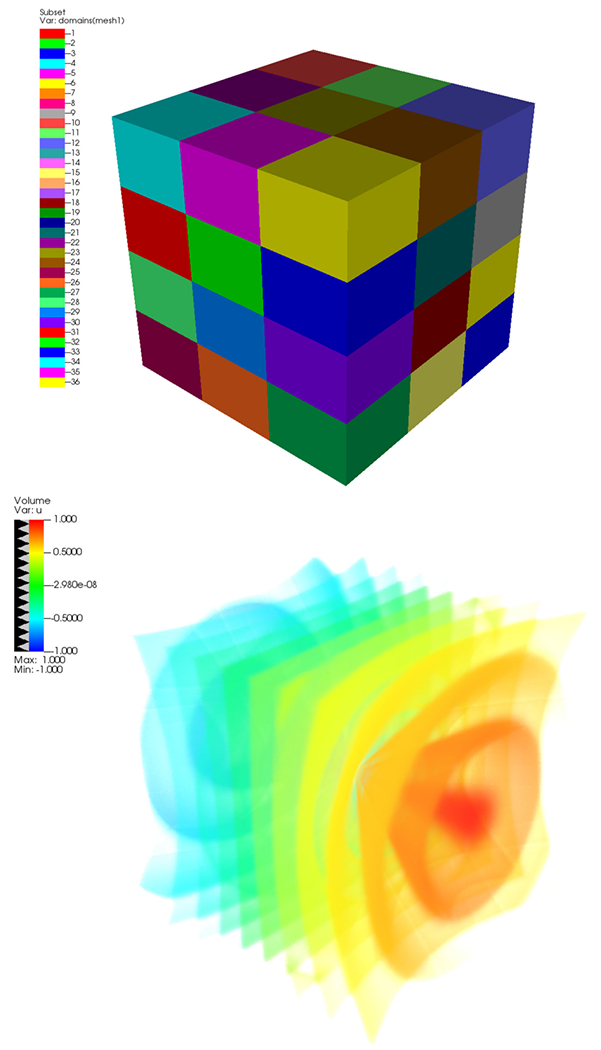
VisIt parallel volume rendering of the multi_rect3d. silo sample data with 36 domains using ANARI with the VisRTX back-end. (Top) The multi_rect3d.silo sample data with 36 domains. (Bottom) Volume rendering of the multi_rect3d.silo data in parallel using 8 MPI processes. Each process is responsible for starting it is own VisIt engine to render a subset of the data (4–5 domains) using ANARI and the VisRTX back-end.

The ANARI SDK was integrated with VisIt using the VisRTX back-end. Figure 6 shows two renderings of the human brain MRI data, courtesy of the Mayo Clinic. The data can be downloaded from VisIt’s MRI tutorial. The top image is a surface rendering of the data using VisIt’s pseudocolor plot. The bottom image is a volume rendering using ANARI and the VisRTX back-end. Figure 7 shows a parallel volume rendering of the multi_rect3d.silo sample data that ships with VisIt. The data are decomposed into 36 domains (top image) that are distributed to multiple processors when VisIt is running in parallel. For the bottom image, VisIt was executed with the command-line argument -np 8, which causes eight MPI ranks to run parallel VisIT engines. Each engine used ANARI, with the VisRTX back-end, to render a subset of the original data (4–5 domains). The partial subimages were then composited into a final image using IceT.^a^

**FIGURE 8.**
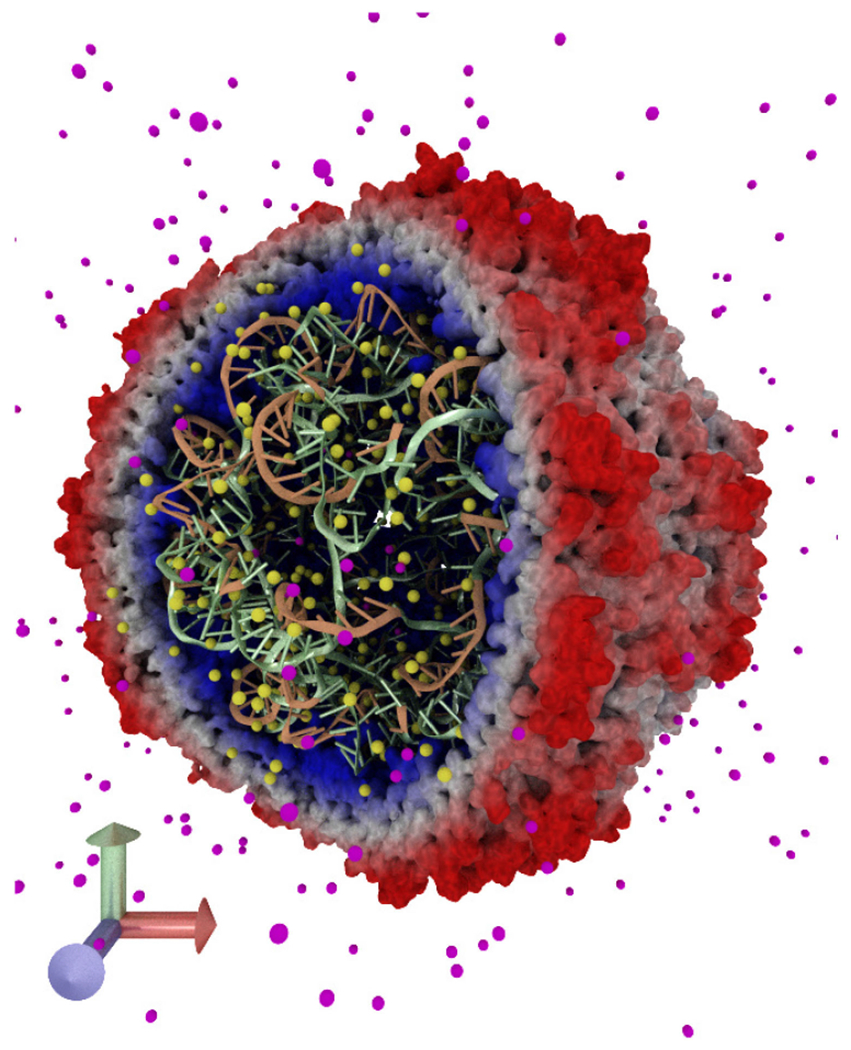
VMD visualization of Satellite Tobacco Mosaic Virus capsid and its interior RNA, with surrounding solvent ions. Rendered from within VMD using the Intel OSPRay ANARI device. ANARI’s support for curved geometric primitives, such as spheres, cylinders, cones, and curves greatly reduces the memory footprint for molecular scenes, and provides the best opportunity for high-quality rendering. Advanced lighting features, such as ambient occlusion, make important biomolecular structures, such as pockets, pores, and cavities immediately visually apparent, making it more intuitive to interpret complex geometric and spatial relationships of molecular components.

**FIGURE 9.**
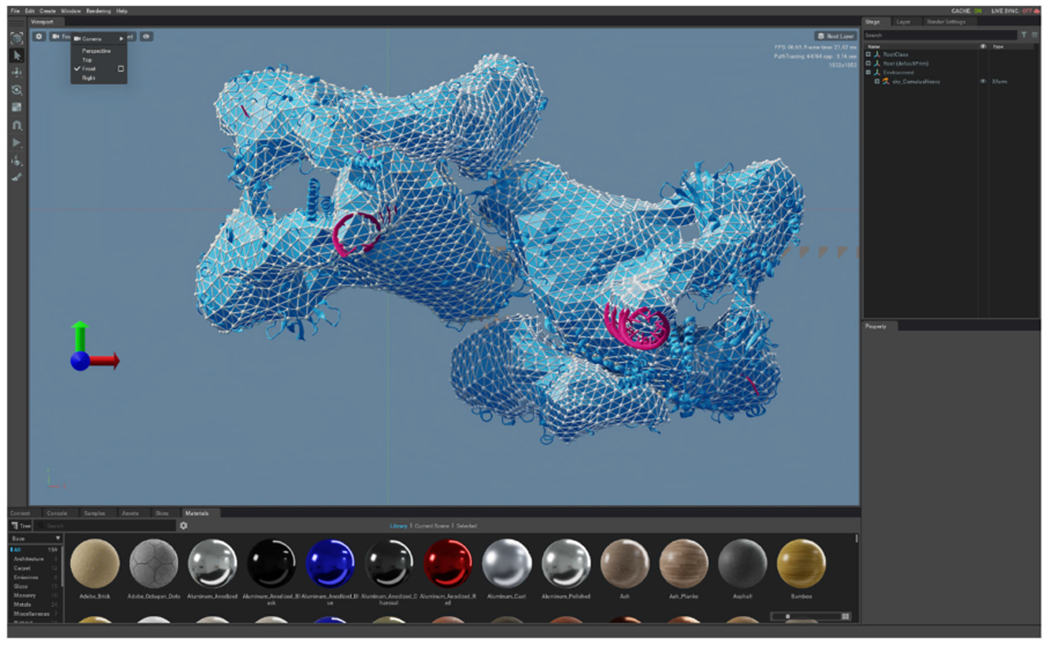
VMD COVID-19 replication transcription complex FFEA tetrahedral mesh visualization imported into NVIDIA Omniverse Create, using an ANARI back-end for the Pixar USD scene format. The USD back-end is capable of both file-based scene export, and live network bridging directly from a visualization application to Omniverse. ANARI “name” label parameters assigned to geometry, groups, and instances ensure that human-readable labels remain associated with the visualization’s scene hierarchy within design, visualization, and rendering tools.

**FIGURE 10.**
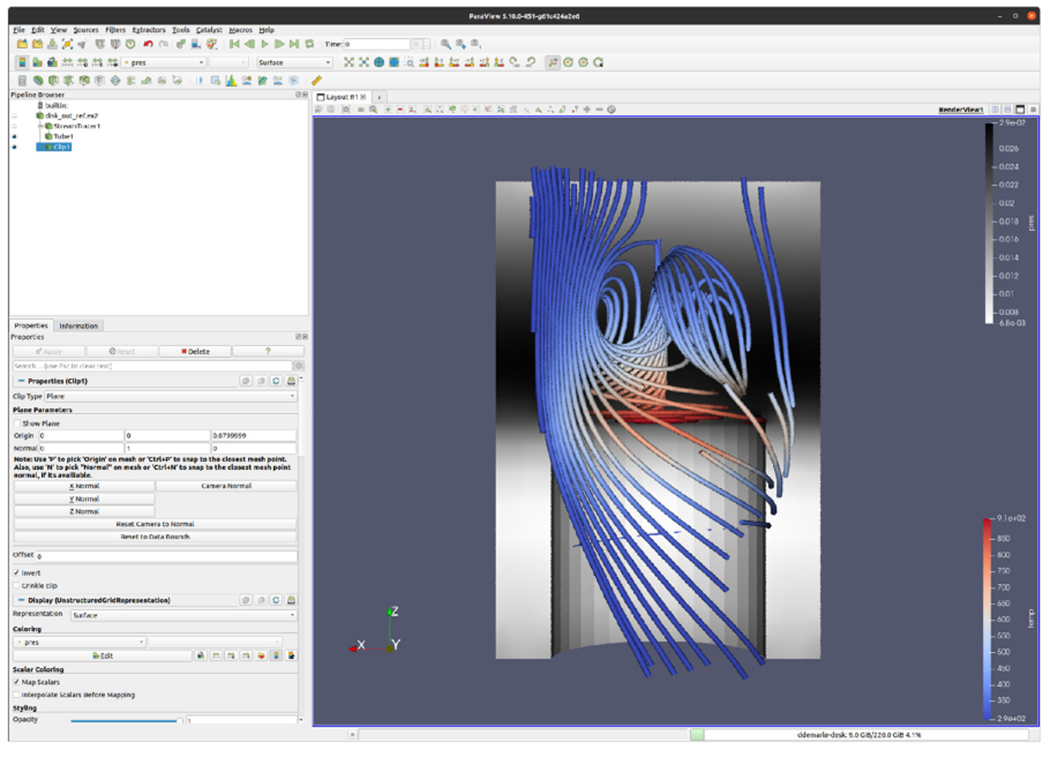
ParaView visualization of the canonical disk_out_ref.ex2 example CFD problem rendered with the ANARI example back-end device.

**FIGURE 11.**
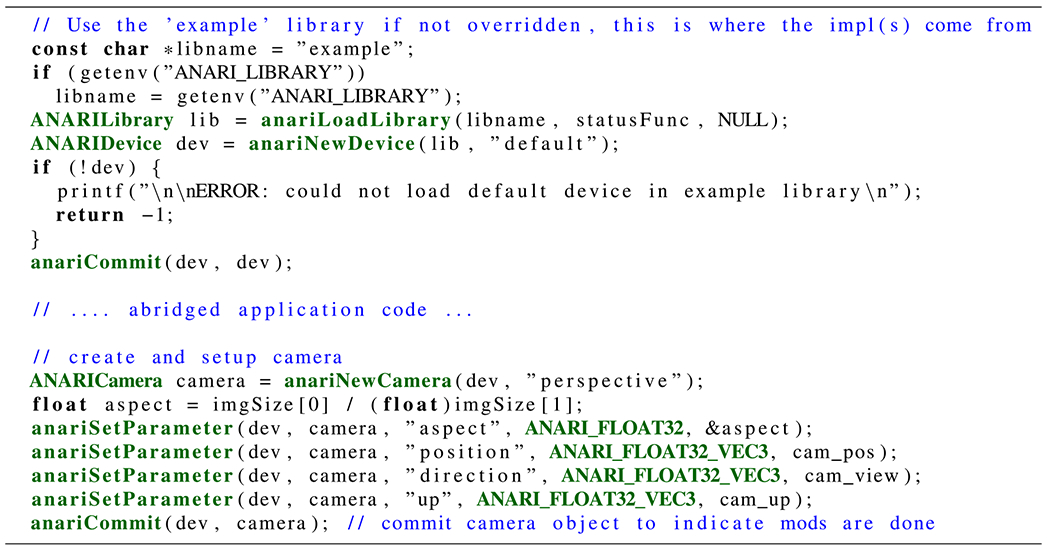
Example ANARI API calls required to load and instantiate an ANARI “device” implementation, and to create a perspective camera, and set its associated parameters.

**FIGURE 12.**
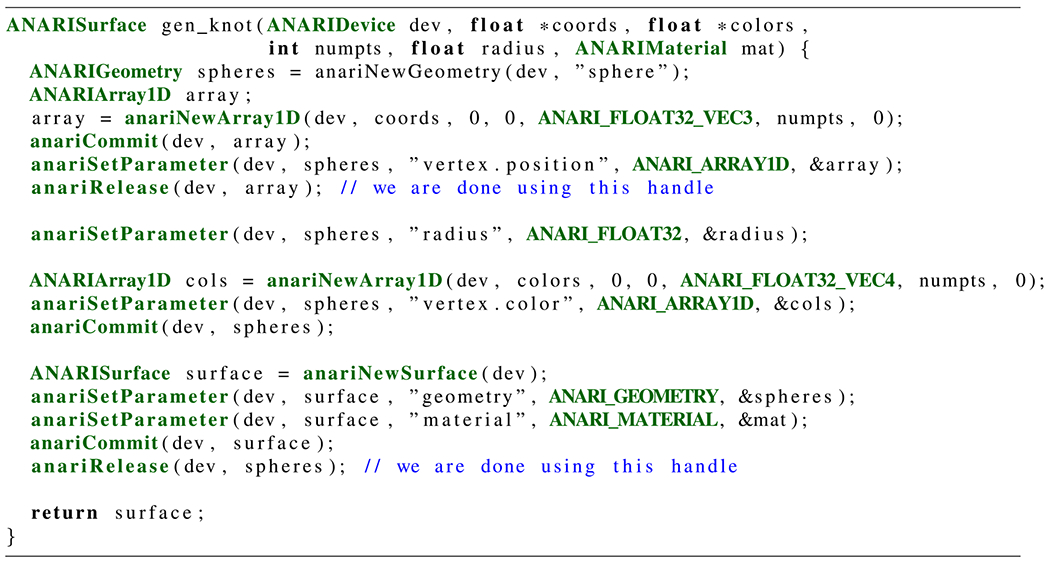
ANARI API calls that add an array of spheres to the scene, adapted from the knot example.

**FIGURE 13.**
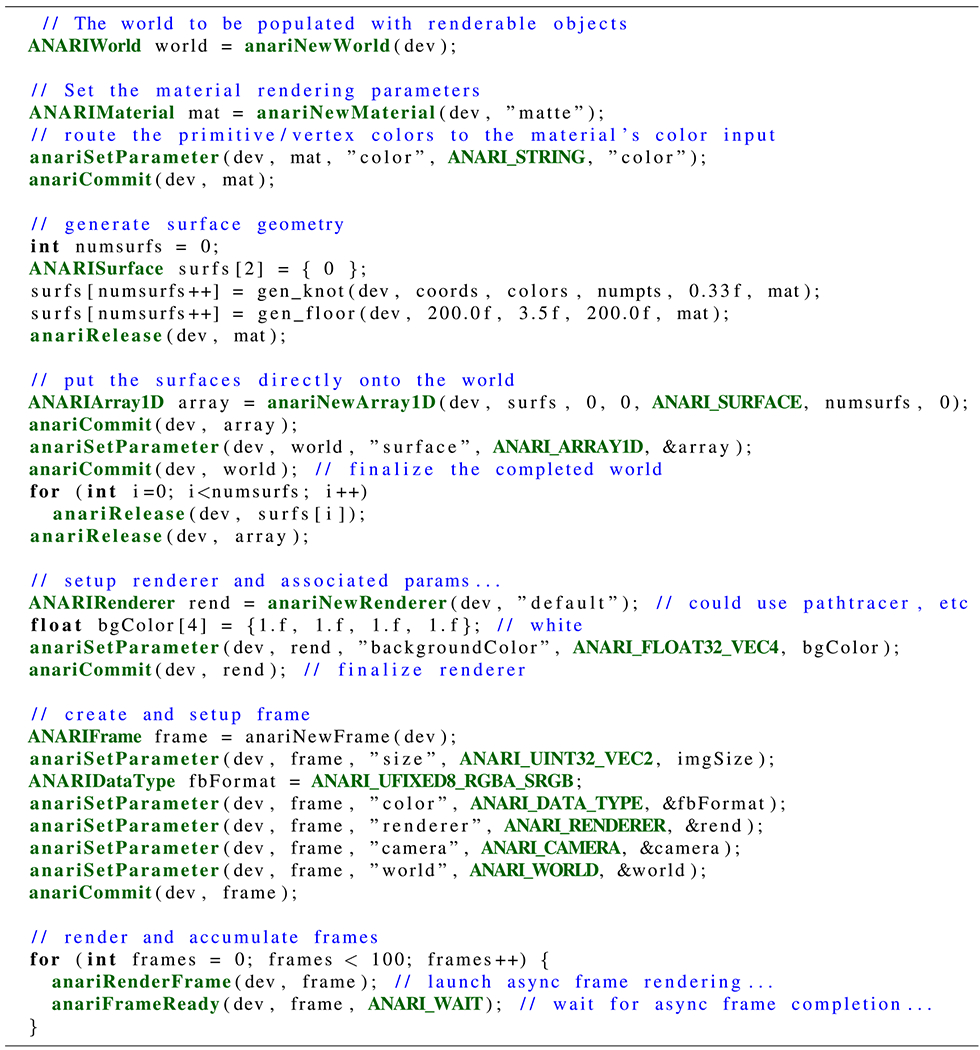
ANARI example scene setup and rendering loop, adapted from the knot example.

A prototype ANARI rendering interface was incorporated into VMD, following the same general structure as VMD’s existing ray tracing engines based on Tachyon, OptiX, and OSPRay. To exercise ANARI in the context of the molecular visualization domain, a variety of existing VMD visualizations were rerendered using developmental ANARI back-end renderer devices, as shown in Figures 8 and 9. ANARI’s support for curved geometric primitives, such as spheres, cones, cylinders, and curves; support for texture mapping and volume rendering; and renderers implementing ambient occlusion lighting and path tracing are all beneficial for molecular visualization.

## CONCLUSION

The goal of ANARI is to create a royalty-free open API standard for cross-vendor access to the state-of-the-art rendering engines. ANARI enables experts in various domains, such as scientific visualization and entertainment, to leverage the latest rendering technology without the need to use low-level rendering APIs. This significantly reduces software development costs while making advanced rendering techniques more accessible and widely used by 3-D visualization applications for which rendering is one of many necessary components in a given software solution. By supporting a well-designed, cross-platform API standard, graphics vendors’ rendering software, and hardware offerings are accessible to a wider diversity of disciplines and audiences.

As an open standard under Khronos Group governance (a nonprofit standards organization), anyone can contribute to development of the ANARI specification as a working group member, or as an external advisor, by contacting the working group.^b,c^ At the time of writing, the ANARI specification has provisional status, and work is focused on finalizing ANARI 1.0. The ANARI SDK and latest specification are publicly available in GitHub and the SDK includes links to available ANARI implementations.^d,e^

## Footnotes

a
https://icet.sandia.gov/

b
https://www.khronos.org/anari

c
https://www.khronos.org/about

d
https://github.com/KhronosGroup/ANARI-SDK

e
https://github.com/KhronosGroup/ANARI-Registry
